# Frizzled related protein deficiency impairs muscle strength, gait and calpain 3 levels

**DOI:** 10.1186/s13023-020-01372-1

**Published:** 2020-05-24

**Authors:** Leire Casas-Fraile, Frederique M. Cornelis, Domiziana Costamagna, Anabel Rico, Robin Duelen, Maurilio M. Sampaolesi, Adolfo López de Munain, Rik J. Lories, Amets Sáenz

**Affiliations:** 1 Biodonostia Health Research Institute, Neurosciences Area, San Sebastian, Spain; 2 Spanish Ministry of Economy & Competitiveness, Carlos III Health Institute, CIBER, Madrid, Spain; 3 Department of Development and Regeneration, Skeletal Biology and Engineering Research Centre, Laboratory of Tissue Homeostasis and Disease, KU Leuven, Leuven, Belgium; 4 Department of Development and Regeneration, Stem Cell Institute, Laboratory of Translational Cardiomyology, KU Leuven, Leuven, Belgium; 5 Department of Public Health, Experimental and Forensic Medicine, Human Anatomy Unit, University of Pavia, Pavia, Italy; 6 Department of Neurology, Donostia University Hospital, Donostia, Spain; 7 Department of Neurosciences, University of the Basque Country, Leioa, Spain; 8 Division of Rheumatology, University Hospitals Leuven, Leuven, Belgium

**Keywords:** LGMD2A, LGMDR1, Calpain 3, *FRZB*, Wnt signalling pathway, Limb girdle muscular dystrophy

## Abstract

**Background:**

Limb-girdle muscular dystrophy recessive 1 calpain3-related (LGMDR1), previously known as LGMD2A, is a disease caused by mutations in the *CAPN3* gene. It is characterized by progressive weakness and muscle degeneration. Frizzled related protein (*FRZB*), upregulated in LGMDR1, was identified as a key regulator of the crosstalk between Wnt and integrin signalling pathways. *FRZB* gene silencing showed a recovery in the expression of some of the costamere protein levels in myotubes.

**Results:**

Here, we performed a comprehensive characterization of *Frzb*^−/−^ mice muscles to study the absence of Frzb in skeletal muscle and eventual links with the molecular characteristics of LGMDR1 patient muscles. *Frzb*^−/−^ mice showed reduced muscle size and strength. Gait analysis showed that *Frzb*^−/−^ mice moved more slowly but no impaired regeneration capacity was observed after muscle injury. Additionally, *Frzb*^−/−^ mice muscle showed an increased number of mesoangioblasts. Lack of *Frzb* gene in *Frzb*^−/−^ mice and its increased expression in LGMDR1 patients, showed contrary regulation of *Rora*, *Slc16a1*, *Tfrc* and *Capn3* genes. The reciprocal regulation of *Frzb* and *Capn3* genes further supports this axis as a potential target for LGMDR1 patients.

**Conclusions:**

Our data confirm a role for *Frzb* in the regulation of *Rora*, *Slc16a1*, *Tfrc*, and *Capn3* genes in muscle cells. In vivo, reduced muscle strength and gait in the *Frzb*^*−/−*^ mice are intriguing features. The reciprocal relationship between FRZB and CAPN3 further supports a key role for this axis in patients with LGMDR1.

Muscular dystrophies are a heterogeneous group of genetic disorders characterized by progressive weakness and muscle degeneration. Among these, Limb-girdle muscular dystrophy recessive 1 calpain3-related (LGMDR1), previously known as LGMD2A, is a disease caused by mutations in the *CAPN3* gene [1]. LGMDR1 was considered the most frequent type of LGMD worldwide [2–5] up till now, although new data suggest that this may not apply to some regions of Latin America [6]. In LGMDR1 patients, disease symptoms caused by muscle wasting and gradual degeneration of the proximal muscle groups, usually first present during the second decade of life and progressively worsen with patients becoming wheelchair-dependent after less than 25 years of evolution of the disease [7, 8]. The pathophysiological mechanisms underlying the process of muscle degeneration in the absence of functional CAPN3 protein are still largely unknown.

Loss of CAPN3 leads to abnormal sarcomere formation [9] as well as changes in the expression of several genes in the muscles of LGMDR1 patients [10]. Sarcomere assembly and stabilisation are dependent on a protein complex called the costamere [11–13]. Its function is to enable the adhesion between the sarcomere in the muscle and the extracellular matrix [14]. This linkage is partially mediated by integrins [15]. In LGMDR1 myotubes, the physiologically required replacement of the integrin β1 isoforms (β1A substitution by β1D) is disturbed and may be the cause of incorrect costamere assembly. Our studies on integrin interacting proteins as well as proteins implicated in costamere regulation, identified frizzled related protein (FRZB) as a key regulator of the crosstalk between integrin and Wnt signalling pathways [16].

FRZB was originally identified as a secreted antagonist of the Wnt signalling pathway, blocking the effects of Wnt-1, Wnt-8, Wnt-5a and Wnt-9a [17–20]. In muscle tissue of LGMDR1 patients *FRZB* gene expression is strongly upregulated [10] and *FRZB* is a negative regulator of myogenesis [21]. Upon silencing of *FRZB* gene expression in LGMDR1 patients’ myotubes, we earlier showed a recovery in the expression of some of the costamere protein levels such as integrin β1D, melusin and anosmin-1 [16].

*Frzb*-deficient mice were shown to be highly susceptible to the development of osteoarthritis characterized by damage to the articular cartilage, and also have increased cortical bone density [22]. Of note, *Frzb*^*−/−*^ mice showed reduced voluntary exercise performance in running wheels [23]. Here, we aimed to further study the effects of absence of *Frzb* in skeletal muscle of *Frzb*^−/−^ mice, and eventual links with the molecular characteristics of LGMDR1 patient muscles.

## Results

### Skeletal muscle analysis

**Fig. 1:**
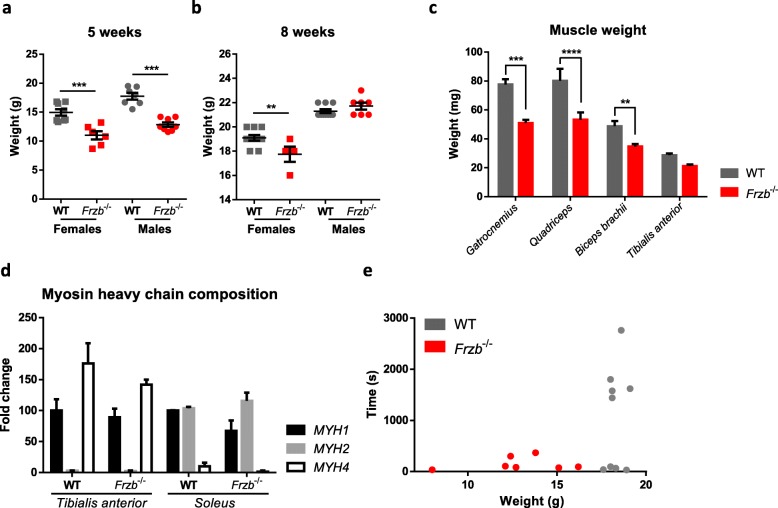
*Frzb*^−/−^ mice are smaller than wild-types (WT) with reduced muscle size and strength. **a-b** Body weight (g) of 5- and 8-week-old WT and *Frzb*^−/−^ mice [*n* = 8 WT-females, 7 WT-males, 6 *Frzb*^−/−^-females and 8 *Frzb*^−/−^-males; *** *p* < 0.0001, p < 0.0001 and ^**^p = 0.01, Bonferroni-corrected for two tests in two-way ANOVA]. **c** Muscle weights (mg) of 4–5-week-old WT and *Frzb*^−/−^ mice [*n* = 15 WT and 14 *Frzb*^−/−^ mice, ^***^ p < 0.0001, **p = 0.009, Bonferroni-corrected for four tests in two-way ANOVA]. **d** Real-time PCR analysis of *Myh1*, *Myh2* and *Myh4* in *Tibialis anterior* and *Soleus* of WT and *Frzb*^−/−^ mice (n = 3 WT and 3 Frzb^−/−^ mice for *Tibialis anterior* and 2 WT and 4 *Frzb*^−/−^ for *Soleus* samples). **e** Muscle strength reflected by hanging time in male WT and *Frzb*^−/−^ mice [n = 9 WT and 7 *Frzb*^−/−^ mice, * *p* = 0.048 for effect of genotype, *p* = 0.8 for effect of weight in two-way ANOVA]. Error bars indicate mean ± SEM

Routine histological analysis throughout our experiments did not reveal any striking differences in muscle structure between wild type and *Frzb*^*−/−*^ mice. At the age of 5 weeks, male and female *Frzb*^*−/−*^ mice were on average 4.92 g [95%CI: 3.09–6.75; *p* < 0.0001] and 3.95 g [95%CI: 2.04–5.86; p < 0.0001] smaller in weight compared to wild-type mice [F(1,25) = 64.2 (*p* < 0.001) - 17.6 (*p* = 0.003) for genotype and sex by two-way ANOVA]. At the age of 8 weeks, these differences persisted only in female mice [difference between means 1.35 g (95%CI: 0.29–2.40; *p* = 0.010) with F (1,27) = 9.4 (*p* = 0.005) for interaction between sex and genotype by two-way ANOVA] (Fig. 1a-b). This weight difference was not observed earlier in *Frzb*^*−/−*^ mice on an outbred Swiss/CD1 background [23].

To determine whether the absence of Frzb in mice affects baseline muscle mass and characteristics, we isolated different muscle groups (G*astrocnemius, Tibialis anterior, Quadriceps* and *Biceps brachii*) from male and female wild-type and *Frzb*^*−/−*^ mice aged 4 to 5 weeks. Muscle weight for the different muscle groups was significantly lower in *Frzb*^*−/−*^ mice compared to wild-type controls. There was a statistically significant interaction between muscle type and genotype on weight [F(1,25)=5.455 (*p* = 0.019) by two-way ANOVA]. The *Gastrocnemius, Quadriceps* and *Biceps* muscle were on average 28.3, 28.02 and 14.98 mg heavier in wild-types compared to *Frzb*^*−/−*^ mice [(95%CI: 18.22–38.41; *p* < 0.001), (95%CI: 17.92–38.11, *p* < 0.0001) and (95%CI: 4.875–25.066, *p* = 0.009), Bonferroni-corrected for four tests in two-way ANOVA] (Fig. 1c). When muscle weight was normalized to body weight, no differences were observed (data not shown) suggesting that the differences may be related to growth retardation.

Earlier analysis in *Frzb*^*−/−*^ mice on the Swiss/CD1 background did not reveal differences in type I vs type IIa fibres in the S*oleus* or in type IIa vs type IIb fibres in the *Extensor digitorum longus* [23]. Here, we further analysed fibre composition at the gene expression level: different myosin heavy chain isoforms (*Myh1*, *Myh2* and *Myh4*) were analysed in 10-week-old male and female *Frzb*^*−/−*^ and wild-type mice. The distinct genotypes did not show differences in the expression of the respective myosins (Fig. 1d). As expected *Myh4* (myosin present in 2B fibre type) followed by *Myh1* (myosin present in 2X fibre type) were highly expressed in the *Tibialis anterior* while *Myh2* showed only minimal expression (myosin present in 2A fibre type). In the *Soleus* expression of *Myh1* and *Myh2* was balanced while *Myh4* was minimally expressed.

To screen for a potential functional impact of these observations, we assessed mouse muscle strength and endurance by the four-limb hanging test. Male *Frzb*^−/−^ mice were performing inferior to wild-type mice in this set-up, independent of weight [difference between means 895.52 s (95%CI: 10.58–1780.47) in 2-way ANOVA with F(1,12) = 4.861; *p* = 0.048 for genotype] (Fig. 1e). Taken together, these analyses suggest that *Frzb*^*−/−*^ mice have reduced muscle size and strength that is not explained by obvious changes in fibre types.

### Gait analysis

**Fig. 2:**
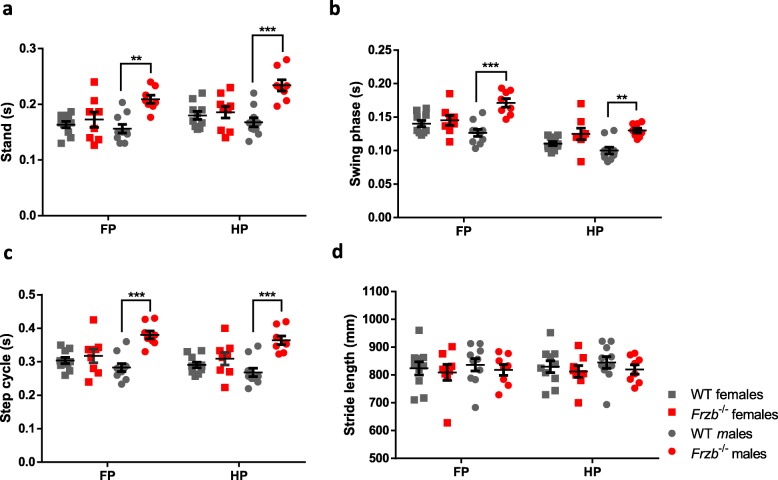
*Frzb*^−/−^ mice moved more slowly with no differences in stride length. **a-d** Gait analysis of 8-week-old female and male wild-type and *Frzb*^−/−^ mice. Average stand, swing phase, step cycle and stride length analysed with ANOVA accounting for genotype (WT, KO), gender (yes, no), paw [front paw (FP), hind paw (HP)] and all interactions. Error bars indicate mean ± SEM. *** p < 0.0001, ^**^p < 0.01 by Bonferroni-corrected test for four tests in two-way ANOVA

We earlier demonstrated that deletion of *Frzb* reduces voluntary running exercise performance in mice using running wheels [23]. We therefore set-out to analyse the gait of the *Frzb*^*−/−*^ mice compared to wild-type controls to evaluate the global mobility of these mice by Catwalk gait analysis [24]. *Frzb*^−/−^ mice paws spent significantly more time in contact with the glass plate and in the air as shown by *stand* and *swing phase* analysis respectively (Fig. 2a-b). There was a statistically significant interaction between the effects of genotype, sex and front- or hind-paw on stand [F(1,34)=4.84, p = 0.04] and swing phase [F(1,34)=12.53, *p* = 0.01] by two-way ANOVA, subsequently Bonferroni corrected post-hoc tests were carried out. For male mice, stand was different between wildtype and *Frzb*^*−/−*^ mice in both front- and hind paws [*p* = 0.002 and *p* < 0.001 respectively], but there was no evidence that the stand differed in females. Male *Frzb*^*−/−*^ mice had on average 0.053 [95%CI: 0.028–0.077] and 0.066 [95%CI: 0.041–0.091] seconds longer stand time in front and hind-paws compared to wildtypes. Similarly, swing was different between male wildtype and *Frzb*^*−/−*^ mice in both front- and hind-paws [p < 0.001 and *p* = 0.005 respectively], but there is no evidence that the stand differed in females. Male *Frzb*^*−/−*^ mice had on average 0.045 [95%CI: 0.029–0.060] and 0.029 [95%CI: 0.014–0.044] seconds longer swing time in front and hind-paws compared to wildtypes. As a consequence, *step cycle* was on average 0.097 s longer in male *Frzb*^−/−^ mice [95%CI: 0.060–0.134, *p* < 0.001] (Fig. 2c). Although *Frzb*^−/−^ mice moved more slowly (increased stand and swing phase), the distance covered by their paws, *stride length*, did not differ between WT and *Frzb*^−/−^ mice (Fig. 2d).

### Treadmill exercise of mice

**Fig. 3:**
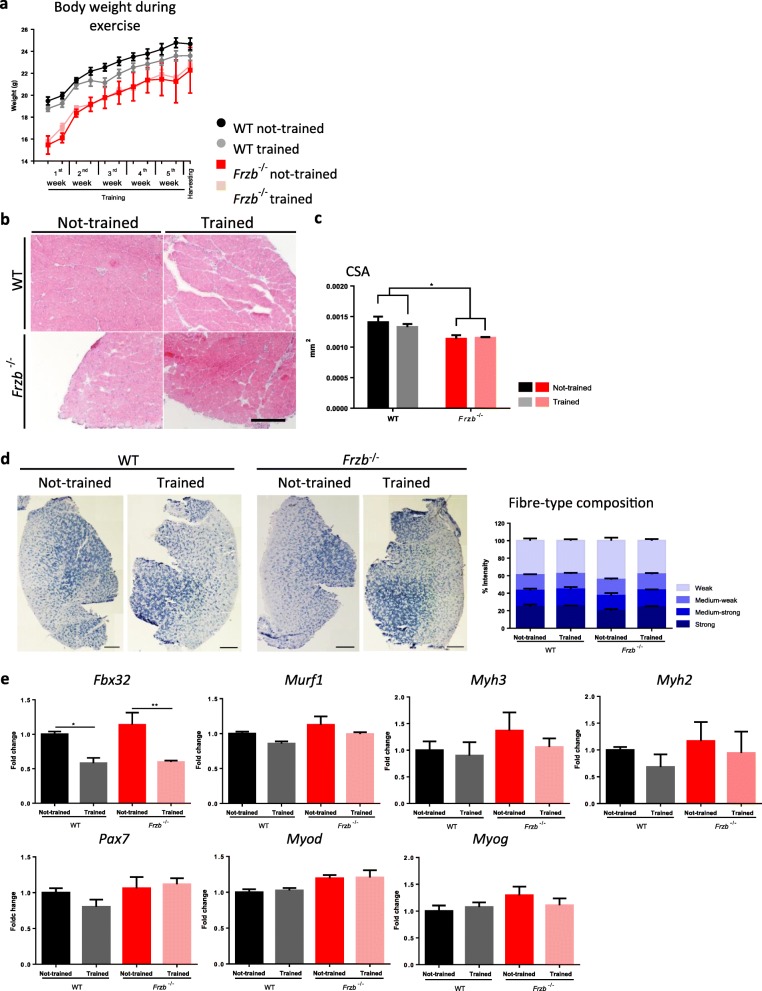
Frzb^−/−^ and wild-type (WT) mice do not respond differently to exercise. **a** Differences between WT and *Frzb*^−/−^ body weight during treadmill exercise [*n* = 4 (WT-not-trained male), 5 (WT-trained male), 2 (*Frzb*^−/−^-not-trained male and female) and 3 (*Frzb*^−/−^-Trained male), *p* < 0.001 for *Frzb*^−/−^ vs WT over time by two-way ANOVA]. **b** Hematoxylin-eosin staining of *Soleus* from WT and *Frzb*^−/−^ mice at the end of the treadmill experiment (scale bar 250 μm) and (**c**) muscle fibre cross-sectional area measurement [n = 4 (WT-not-trained male), 5 (WT-trained male), 3 (*Frzb*^−/−^-not-trained 1 male and 3 female) and 3 (*Frzb*^−/−^-Trained male)] * *p* = 0.0066 for genotype by two-way ANOVA]. **d** SDH staining of *Tibialis anterior* from WT and *Frzb*^−/−^ at the end of the experiment and fibre type quantification. **e** Real-time PCR analysis of *Fbx32*, *Murf1*, *Myh3*, *Myh2*, *Pax7*, *Myod* and *Myog* in *Gastrocnemius* of WT and *Frzb*^−/−^ mice (n = 3 male for each group). Error bars indicate mean ± SEM

To document whether *Frzb*^−/−^ and wild-type mice react differently to exercise, animals were subjected to treadmill running. All animals in the experiment were able to perform the selected chronic running exercise protocol and no abnormal behaviour or signs of exhaustion were observed. First, mice body weights were measured, differences between WT and *Frzb*^−/−^ body weight were gradually diminishing over time, and no differences between trained and not-trained groups were seen [2-way ANOVA (F (11,41) = 5.850 (*p* < 0.001) for interaction age and genotype, F([11,41) = 0.972 (*p* = 0.97) for interaction age, genotype and running] (Fig. 3a). Fibre cross-sectional area of S*oleus* was measured. Although no overall differences between cross-sectional areas were observed between trained and not-trained groups, *Frzb*^−/−^ mice fibres were smaller than WT fibres, most notable in the not-trained group [2-way ANOVA (F(1,11) = 11.13 (*p* = 0.0066), 0.51 (*p* = 0.4865), 0.22 (*p* = 0.64) for genotype, running and interaction respectively, difference between means 2.7*10^− 4^ mm^2^(95%CI: 2.05*10^− 5^ – 5.2*10^− 4^; *p* = 0.034) and 1.7*10^− 4^ mm^2^ (95%CI: − 6.46*10^− 5^ – 4.1*10^− 4^; *p* = 0.17) Bonferroni-corrected for 2 tests in non-trained and trained mice] (Fig. 3c). No exercise-induced damage was observed in any of transverse sections of S*oleus* or *Tibialis anterior* (Fig. 3d). Muscle fibre type composition was analysed in both study groups in *Tibialis anterior* muscles and no differences were observed after exercise or between genotypes (Fig. 3d). Gene expression markers for muscle damage were not changed by exercise or genotype, with *Fbx2* as a positive control for the effect of training (difference between the means 44.67, 95%CI: 23.49–65.85) (Fig. 3e).

### Muscle regeneration capacity after intramuscular cardiotoxin-induced muscle injury

**Fig. 4:**
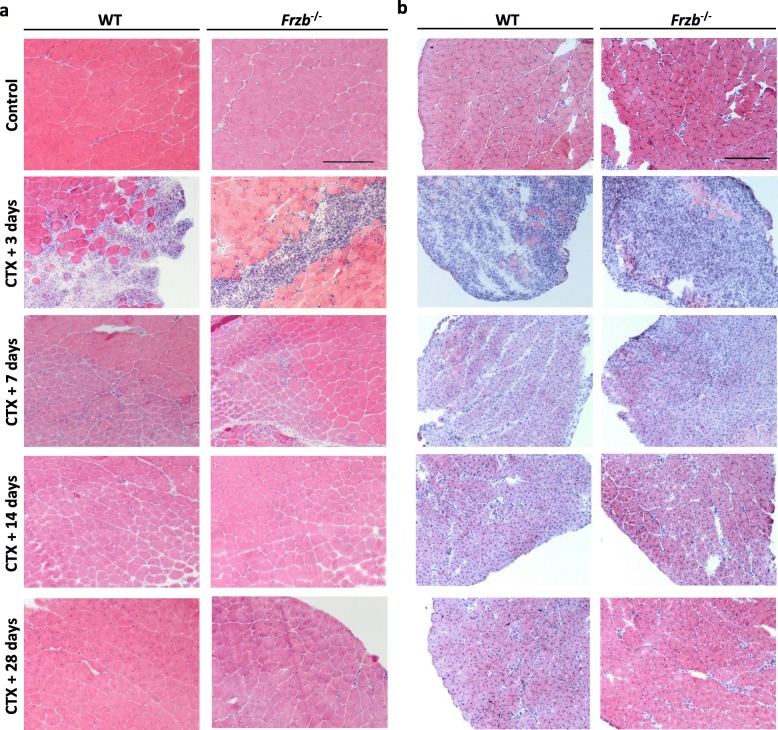
Cardiotoxin (CTX) injection trigger no differences in damage between Frzb^−/−^ and wild-type (WT) mice. Hematoxylin-eosin staining of (**a**) *Tibialis anterior* and (**b**) *Soleus* sections of 10-week-old female and male WT and *Frzb*^−/−^ mice after 3, 7, 14 and 28 days after cardiotoxin injection. Scale bar (**a**) 250 μm and (**b**) 50 μm

To test whether muscle regenerative capacities of *Frzb*^*−/−*^ mice were different from wild-type mice, acute *skeletal muscle* injury was triggered by *cardiotoxin* injection in the S*oleus* and *Tibialis anterior* muscles. No differences in pathology characteristics were noted between the two mouse strains. Non-injured skeletal muscle shows polygonal myofibres with peripheral nuclei (Fig. 4a-b). At day 3 post injection, muscles showed degenerative myofibres and inflammatory cellular infiltration. In the *Soleus*, most of the fibres were damaged, while in the case of *Tibialis anterior,* just the cardiotoxin injection area appeared affected (Fig. 4a-b). One week after injury, small regenerating myotubes with centrally located nuclei were observed with some remnants of inflammation. Regenerating fibers with centrally located nuclei increased their diameter by two weeks and the pattern became homogenous by four weeks (Fig. 4a-b).

### In vitro muscle cell differentiation

**Fig. 5:**
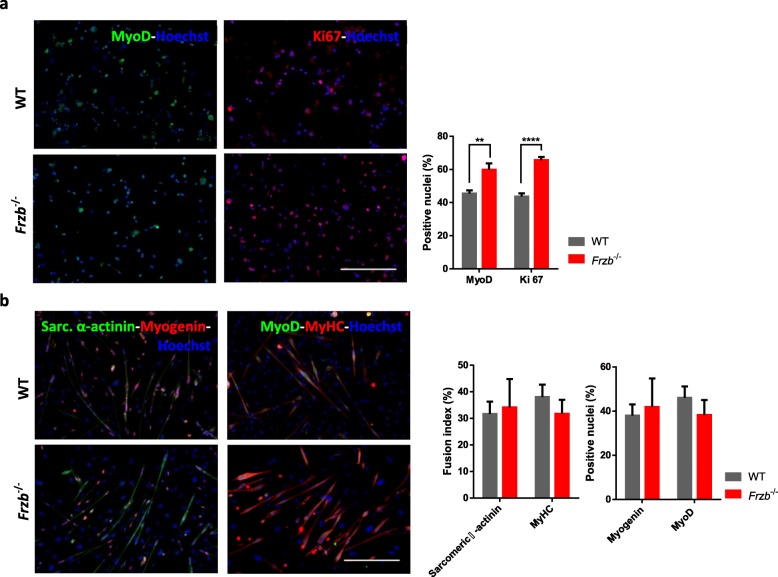
*Frzb*^−/−^ mice have increased MyoD and Ki67 positive satellite cells but no detectable differences in differentiation capacity. **a** Immunofluorescence analysis of satellite cells extracted from 4-week-old WT and *Frzb*^−/−^. Left images stained for MyoD (green) likewise right images for Ki67 (red). Percentage of MyoD and Ki67 positive nuclei [n = 7 (WT 3 male and 4 female) and 6 (*Frzb*^−/−^ 3 male and 3 female), *P* = 0.0046 and *P* < 0.0001 respectively, t-test]. **b** Immunofluorescence analysis of WT and *Frzb*^−/−^ myotubes at day 3 of differentiation. Left images stained for nuclear myogenin (red) and cytoplasmatic sarcomeric α-actinin (green). Right images stained for nuclear MyoD (green) and cytoplasmatic MyHC (red). Myotubes fusion index, calculated as the percentage of nuclei inside myotubes on the total amount of nuclei. In all cases nuclei were visualized with Hoechst (blue). Scale bar 250 μm. Error bars indicate mean ± SEM

Taking into account that Frzb affects myogenesis and that myotube differentiation and costamere assembly appear disturbed in LGMDR1 patients and models, we studied muscle cell differentiation in the different genetic backgrounds, using two different cell populations with myogenic potential. First, we isolated satellite cells from four-weeks-old wild-type and *Frzb*^*−/−*^ mice using the *Biceps brachii, Gastrocnemius, Tibialis anterior* and Q*uadriceps.* In proliferation assays, immunofluorescence analysis showed more MyoD positive cells in *Frzb*^−/−^ mice muscles after enzymatic digestion and increased levels of the proliferation marker protein Ki67, represented as a percentage [*P* = 0.0046 and *P* < 0.0001 respectively, t-test] (Fig. 5a). However, after differentiation was induced, at the myotube stage fusion index and numbers of MyoD and myogenin positive nuclei were not different between the wild-type and *Frzb*^*−/−*^ mice (Fig. 5b).

**Fig. 6:**
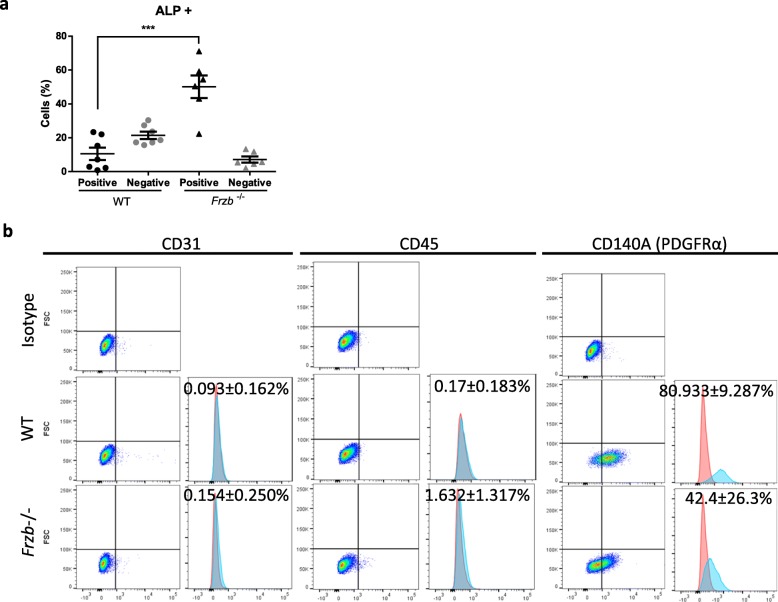
*Frzb*^−/−^ mice have an increased percentage of ALP-positive mesangioblasts compared to wild-type (WT) animals. **a** Percentage of alkaline phosphatase positive and negative cells [n = 7 (WT 4 male and 3 female) and 6 (*Frzb*^−/−^ 4 male and 2 female), *** *p* < 0.001 Bonferroni-corrected for two tests in two-way ANOVA]. Error bars indicate mean ± SEM. **b** FACS data showing CD31, CD45 and CD140A expression with mean ± standard deviation [n = 3 (WT male) and 7 (*Frzb*^−/−^ 4 male and 2 female), *p* = 0.043 for CD140A, t-test]

In addition, we isolated mesoangioblasts (MABs) from five-week-old wild-type and *Frzb*^*−/−*^ mice using the *Biceps brachii, Gastrocnemius, Tibialis anterior* and Q*uadriceps* as source. Freshly isolated cells were analysed by FACs analysis using alkaline phosphatase (ALP) as a cell surface marker. The obtained ALP+ cell distribution was different between wild-type and *Frzb*^−/−^ mice. Percentage of ALP+ cells from *Frzb*^*−/−*^ mice was on average 39.59 higher than from wild-type animals [95%CI: 26.18–53.00; *p* < 0.0001), Bonferroni corrected for 2 tests in 2-way ANOVA with F(1,22) = 46.73 (*p* < 0.001) for interaction between genotype and cell type] (Fig. 6a). Further characterization of the ALP+ cells showed that all of them were indeed negative for endothelial cell marker CD31 and hematopoietic cell marker CD45. Nevertheless, while wild-type ALP+ cells were around 81% positive for platelet derived growth factor receptor alpha (CD140A or PDGFRα), *Frzb*^−/−^ ALP+ cells were only around 43% positive for this marker [*p* = 0.043 for CD140A, t-test] (Fig. 6b).

### Molecular analysis of the muscles

**Fig. 7:**
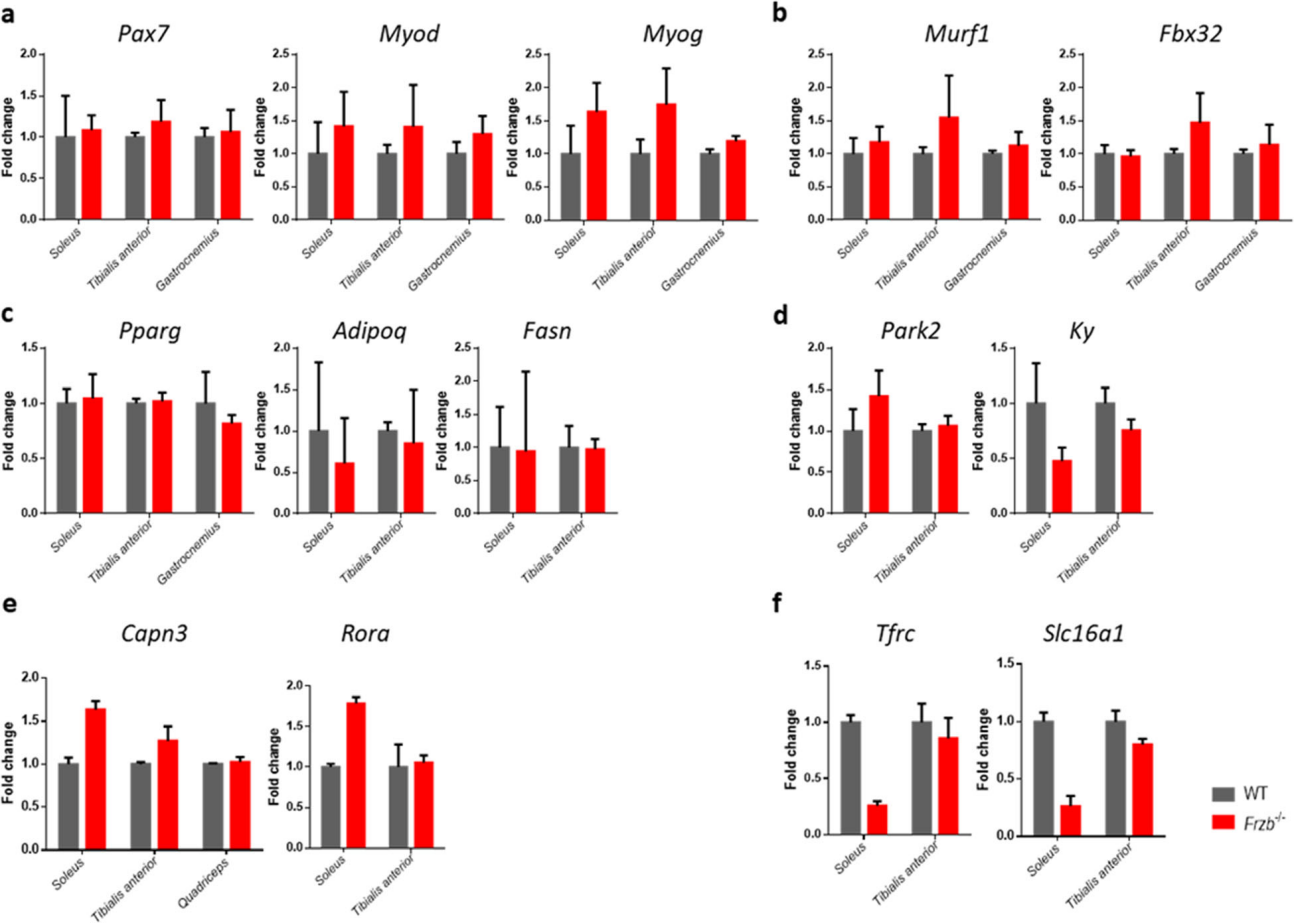
Real-time PCR analysis of *Tibialis anterior* and *Soleus* of 10-week-old wild-type (WT) and *Frzb*^−/−^ mice. **a** muscle specific genes *Pax7*, *Myod* and *Myog* (> 0.05, *p* = 0.005 and 0.06 respectively), (**b**) muscle atrophy-related ubiquitin ligases *Fbx32* and *Murf1* (all *p* > 0.05), (**c**) adipose tissue related genes, *Pparg*, *Adipoq* and *Fasn* (all p > 0.05), (**d**) genes deregulated in *Capn3*^−/−^ mice, *Park2* and *Ky* (*p* = 0.05 and 0.0024), (**e**) upregulated genes in *Frzb*^−/−^ mice, *Capn3* and *Rora* (*p* = 0.014, 0.0025) and (**f**) genes differentially expressed in muscles from LGMDR1 patients and in the articular cartilage of *Frzb*^−/−^ mice, *Tfrc* and *Slc16a1* (*p* = 0.0005, 0.007). Error bars indicate mean ± SEM

To screen for molecular differences between the muscles of *Frzb*^−/−^ and wild-type mouse muscles and find eventual links with LGMDR1, we performed gene expression analysis on isolated muscles. First, we focused on muscle-specific genes. On one hand, no differences were found for *Pax7*. However the expression of *Myod* was on average 52.73% higher in *Frzb*^−/−^ mouse muscles compared to wild types [(95%CI: 18.18–87.33) by Mixed Effect analysis with F(1, 17)= 10.34 (*p* = 0.005) for genotype]. A similar trend was observed for myogenin [(95%CI: − 1715–76.59] by Mixed Effect analysis with F(1, 17)= 4.07 (*p* = 0.06) for genotype]. (Fig. 7a). Muscle atrophy-related ubiquitin ligases *Fbx32* and *Murf1* did not show differences between wild-type and *Frzb*^−/−^ mice (Fig. 7b). The analysis of adipocyte genes *Pparg*, *Adipo* and *Fasn,* showed no differences between wild-type and *Frzb*^−/−^ mice (Fig. 7c).

We then focused on genes that are differentially expressed in *Capn3*^−/−^ mice compared to wild-type mice [25]. *Park2* expression was on average 17.93% higher in *Frzb*^−/−^ mice compared to wild-types [(95%CI: − 8 – 43.68) by Mixed Effect analysis with F(1, 7)= 5.29 (*p* = 0.05) for genotype]. *Ky* expression was on average 35.21% lower in *Frzb*^−/−^ mice [(95%CI: 15.15–55.77) by Mixed Effect analysis with F(1, 12)= 4.26 (*p* = 0.0024) for genotype] (Fig. 7d). These differences were most pronounced in the *Soleus* muscles.

Other genes of interest were identified from the analysis of LGMDR1 patient muscles with *CAPN3* mutations. Interestingly, *Capn3* expression was on average 63.76% increased in the *Soleus* muscle of *Frzb*^−/−^ mice compared to wildtypes [(95%CI: 21.15–106.4) by t-test with *p* = 0.0142] but not different in *Tibialis anterior* or *Quadricipes* muscles. This was also the case for the *Rora* gene [79.17% increased (95%CI: 46.76–111.6) by t-test with *p* = 0.0025] in the *Soleus* but not in the *Tibialis anterior* (Fig. 7e). On the other hand, *Tfrc* and *Slc16a1* gene expression were 74.26% [95%CI: 54.34–94.17] and 73.67% [95%CI: 33.63–113.7] downregulated in the *Soleus* of *Frzb*^*−*/−^ mice compared to wildtypes [*p* = 0.0005 and *p* = 0.0069 by t-test] (Fig. 7f). Again, no differences were found in the *Tibialis anterior* muscles. Of note, *Tfrc*, *Rora* and *Slc16a1* were not only differentially expressed in muscles from LGMDR1 patients but also in the articular cartilage of *Frzb*^*−*/−^ mice [10, 26].

### FRZB silencing in human muscle cells

**Fig. 8:**
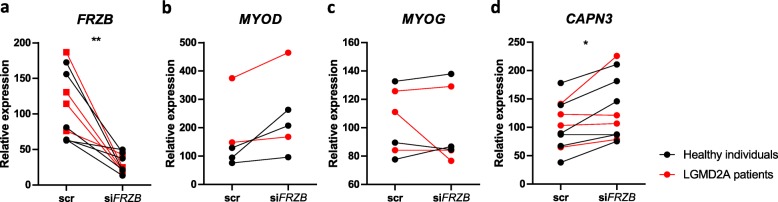
Real-time PCR analysis of healthy individuals and LGMDR1 patients’ myotubes at day 10 of differentiation in *FRZB* silencing experiments. **a***FRZB* gene expression. **b***MYOG* and (**c**) *MYOD* gene expression. **d***CAPN3* gene expression. Expression levels are relative to housekeeping gene *GAPDH*, * *p* = 0.0168 ** *p* = 0.0011 by two-way ANOVA

The effect of specific genes of interest were translationally validated in human myotubes by silencing the expression of the human *FRZB* gene. As shown in Fig. 8a, silencing of *FRZB* was successful in both control and LGMDR1 patient myotubes. On average *FRZB* expression was 84.33% reduced [(95%CI: 44.99–123.7) by two-way ANOVA with F(1,8)=24.4 for silencing (*p* = 0.0011)]. Myogenic markers *MYOD* and *MYOG* were not different between *FRZB* silencing and control conditions (Fig. 8b-c). *CAPN3* gene was on average 28.22% upregulated after *FRZB* silencing [(95%CI: 6.61–49.82) by two-way ANOVA with F(1,8)=9.07 (*p* = 0.0168) for silencing] but the effect was not different between LGMDR1 patients and the healthy donors (Fig. 8d).

## Discussion

The potential role of FRZB in LGMDR1 is intriguing as patients have high levels of FRZB expression in their muscles [10]. In myotubes of LGMDR1 patients, correct costamere assembly and cell fusion appears to be disturbed and this has been linked with the absence of the required integrin isoform replacement from β1A to β1D. Remarkably *FRZB* silencing in myotubes leads to costamere protein rescue. We therefore suggested that FRZB may be a potential therapeutic target for LGMDR1 patients [16]. To better understand the function of endogenous FRZB in muscles, different aspects of muscle biology in the *Frzb*^*−/−*^ mouse model were studied at the functional, cellular and molecular level in an exploratory analysis.

Muscle weakness is the hallmark of muscular dystrophies. Our analysis suggests that *Frzb*^−/−^ mice have a lower muscle mass that may result in reduced performance in the four limb hanging test. These findings seem to correspond to a stunted growth since no differences in body weight were previously observed in 6-month and 1-year old mice [23]. We have no evidence that weakness would evolve over time as this parameter was only evaluated at one time-point.

We also performed gait analysis of *Frzb*^−/−^ mice to gain insights into the effect of *Frzb* deficiency on muscle function. *Frzb*^−/−^ mice showed a longer step cycle, spending more time with the paw in contact with the walkway (stand time) as well as airborne (swing time). Thus, their limb movement was slower, however with no effect on the covered distance. In a previous report, we showed that *Frzb*^−/−^ mice ran daily significantly lower distances in a voluntary running wheel setup [23]. This reduced voluntary running exercise performance could be attributed to the lower speed rather than to the fact that they spend less time running. Thus, *Frzb*^−/−^ mice appear to have detectable issues in speed and mobility compared to wild-type controls. Although interesting from a scientific point of view, these observations cannot be translated into clinically meaningful data that could be applied to dystrophy patients. Anomalies that can be noted in muscular dystrophy patients are linked with weakness of the hip abductor muscles, producing a Trendelenburg gait characterized by “waddling” [27] and differ from what was observed in *Frzb*^−/−^ mice. In addition, the instability and altered step patterns observed among LGMD patients that have been already characterized in a mouse model [27] are different to what was observed in this study.

To better understand the reasons why gait and muscle strength abnormalities were observed, several other factors were analysed. Jackson and collaborators (2015) showed that depletion of *Pax7* expressing satellite cells in muscles resulted in reduced voluntary wheel running performance. Hence, *Pax7* expression was analysed. No differences with wildtype animals were shown. Increased adipose tissue infiltration of muscles is a common hallmark in neuromuscular disorders [28–30]. *Frzb*^−/−^ mice have lower body and muscle mass. However, *Frzb*^−/−^ mice did not show fatty infiltration, centrally located nuclei, fibrosis or other dystrophic features in the analysed muscle sections. Regulators of adipocyte differentiation, peroxisome proliferator activated receptor gamma or adiponectin coded by *Pparg* and *Adipoq* respectively were not different between the studied mouse strains. In addition to fat infiltration, atrophy can also cause muscle weakness, by upregulation of atrogenes (MAFbx/Atrogin-1 and MuRF1) that lead to loss of muscle mass [31–33]. None of the atrogenes were up-regulated in *Frzb*^−/−^ muscles. Therefore, we have no evidence that fat or atrophy could be the reason why mice spent more time to complete a step cycle.

Gait and other phenotypes reported here might not only be altered by impairment in muscles; but also by changes in the nervous system or bones. So far, there is no clear evidence of alterations in the nervous system of *Frzb*^−/−^ mice. Some reports confirm dynamic expression of *Frzb* in neural cells but we are not aware of specific data on motor neurons, motor cortex or cerebellum that would explain our observations [34–38]. Nevertheless, the transgenic mice are known to have thicker cortical bone, with increased stiffness and higher cortical appositional bone formation after loading of the long bones [22]. These differences with wild-type animals cannot be excluded as factors contributing to the changes observed in *Frzb*^−/−^ mouse gait and other phenotypes.

Exercise has direct effects on muscles, triggering changes in CSA of the fibres, fibre type distribution, weight change and potentially muscle injury [39]. Although physical training induces beneficial adaptive changes in skeletal muscle of healthy individuals, its effects in patients with muscular dystrophy remain controversial. While some studies attributed a beneficial effect without reporting muscle injury, other studies reported training-induced muscle damage and creatine kinase elevations in high-intensity training programs in patients, or even an earlier onset of symptoms associated with exercise in LGMD2B patients [40–44]. Since distinct muscular dystrophies show different progression of muscle degeneration and strength loss, leading to diverse exercise tolerance, endurance treadmill training tolerance and muscle changes were studied in *Frzb*^−/−^ model. The lack of structural changes in *Frzb*^−/−^ mice after exercise suggests the absence of a severe phenotype.

The CSA of muscle fibres in *Frzb*^−/−^ mice was smaller. Consequently, we studied muscle fibre type composition. *Tibialis anterior* and *Soleus* fibre type distribution were within normal range values as has been described for C57Bl/6 mice [45, 46]. Previous studies in *Soleus* and *Extensor digitorum longus* analysed by immunofluorescence showed, similar fibre composition in WT and *Frzb*^−/−^ mice [23]. Additionally, myosin gene expression as well as SDH activity were analysed obtaining the same result: fibre composition in *Soleus* and *Tibialis anterior Frzb*^−/−^ did not vary from wild-type mice.

Several muscular dystrophy models, such as syntrophin α1 null mice and murine models for LGMDR12 (LGMD2L) and LGMDR1, showed aberrant muscle regeneration with longstanding necrosis and impaired exercise and contractile properties with aberrant neuromuscular junctions [47–49]. However, in *Frzb*^−/−^ mice, after cardiotoxin injection, no aberrant or impaired regeneration capacity or fibrosis was noticed. In summary, *Frzb*^−/−^ mice muscles showed normal fibre composition and they do not display altered regeneration capacity.

Murine primary cell cultures have been widely studied for myogenesis and muscular dystrophies in vitro analysis such as LGMDR1, LGMD2I or DMD [9, 16, 50–55]. We isolated satellite cells and we found that *Frzb*^−/−^ mice cells showed enrichment for MyoD and Ki67 nuclear proteins. FRZB inhibits MyoD expression at RNA and protein level [21, 56]. We here show that in the absence of FRZB, MyoD was upregulated in our cells. As myotube formation was not altered, myogenesis may not be strongly impaired, but further studies will be required to identify the consequences of MyoD during myogenesis in the absence of FRZB.

On the other hand, the increased Ki67 expression, which is a proliferation marker, suggested an increased proliferative capacity in *Frzb*^−/−^ muscles. However, there is controversy about the way in which the presence or absence of FRZB could affect proliferation. Some studies described that FZRB inhibits the growth of mesoangioblasts and suppressed cell proliferation in gastric cancer [57, 58], but other authors suggested that Frzb suppression reduced proliferation in alveolar rhabdomyosarcoma [59]. Moreover, tissue dependent differences have been observed in the same model, since *Frzb*^−/−^ mice chondrocytes proliferated less than those obtained from WT mice*,* contrary to the observation in satellite cells in the same mice.

Considering that some muscle resident cell population are able to generate muscle, both in vitro and in vivo [60–62] pericyte-derived adult MABs were also studied. Pericyte–derived adult MABs are isolated from adult muscles and they retain similar characteristics of embryonic MABs [63–66]. Detailed analysis of the ALP+ cells showed significantly less PDGFRα expression. So far, two types of pericytes have been described: type-1 and type-2. Both of them differ in their cell surface markers as well as in their differentiation capacity. Type-1 are Nestin−/PDGFRα+ and are characterized by their ability to differentiate into adipocytes while type-2 are Nestin+/PDGFRα- and do not differentiate into adipocytes but form myotubes in culture [67, 68]. The lower PDGFRα expression could indicate that *Frzb*^−/−^ mice have more type-2 pericytes.

In previous studies, differentially expressed genes have been analysed in the articular cartilage-subchondral bone biomechanical unit of *Frzb*^−/−^ mice [22, 26]. However, gene expression analyses in muscle have not been carried out. Thus, one aim of this study was to establish whether *Frzb* deficiency impairs muscle gene expression in *Frzb*^−/−^ mice. The analysis was focused mainly on the *Soleus*, as *Soleus* showed the greatest molecular similarities to human skeletal muscles [69] and since together with diaphragm these are the most affected muscles in *Capn3*^−/−^ mice [9].

Myogenesis, a process that takes place during growth and regeneration in adult, depends on satellite cell activation of Pax7 cells and it is regulated by muscle-specific transcription factors such as MyoD and Myogenin [70, 71]. In the studied samples *Pax7* was not upregulated, *MyoD* was upregulated in *Frzb*^−/−^ and there was some increase in *Myog.* The same was observed in the *FRZB* silenced human samples; *MYOD* expression was upregulated. In Xenopus, Frzb inhibits axis duplication induced by Xwnt8 and also muscle development by blocking MyoD induction [18, 56, 72]. In mammals, myogenesis inhibition by *Frzb* accompanied by reduction in *Myf5* and *MyoD* expression was reported [21]. Although most of the works were carried out during embryonic development, the possibility that *Frzb* has a role in adult myogenesis or muscle maintenance, modualting MyoD levels, should be considered. Although *Myod* was upregulated in *Frzb*^−/−^ mouse muscles, increased myogenesis was not observed (centrally located nuclei were absent and different size fibres were not observed). MyoD increase after si*FRZB* in LGMDR1 patients could be considered as a beneficial consequence given that muscle degeneration stimulus is occurring and consequently new myofibres formation would be necessary. Nevertheless, further studies will be required to analyse if this increase improves cell physiology.

Among the selected genes deregulated in C3KO mice [25] the *Ky* gene showed expression changes in *Frzb*^−/−^ mice. Its protease activity targets different proteins and its absence could disrupt muscle cytoskeleton homeostasis [73]. Natural *ky* mutant mice has smaller muscles with slower contraction time and are weaker than controls [74, 75]. When focusing on deregulated genes in LGMDR1 patients, it is noteworthy to mention that *Rora*, *Slc16a* and *Tfrc* genes showed the same direction of differential expression in cartilage and muscle of *Frzb*^−/−^ mice. These differences were opposite to what was observed in LGMDR1 patients where *FRZB* is upregulated.

Tfrc is implicated in muscle biology. On one hand, it participates in iron acquisition in skeletal muscle [76]. On the other hand, *Tfrc* has been already described as a Wnt target gene [77]. *Tfrc* is elevated in regenerating fibres in patients with Duchenne muscular dystrophy as well as in facioscapulohumeral muscular dystrophy (FSHD) [78, 79]. However, the effects of *TFRC* upregulation in LGMDR1 patients have so far not been studied.

In skeletal muscle, retinoic acid receptor-related orphan receptor-α (Rorα) has been described as positive regulator of myogenesis by its interaction with MyoD and p300 cofactor which lead to activate muscle-specific genes transcription [80]. Furthermore Rorα is involved in the regulation of glucose and lipid metabolism in skeletal muscle [81, 82]. On the other hand, a role in Wnt signalling has been described since Wnt5a/PKCα-dependent as well as PGE2/PKCα-dependent Rorα phosphorylation exerts inhibitory function of the expression of Wnt / β-catenin target genes [83, 84]. Altogether, rescue of *Rora* expression by Wnt signalling pathway activation in the absence of FRZB could be beneficial for LGMDR1 patients due to its importance in muscle homeostasis.

*Slc16a1*, coding for a proton-linked monocarboxylate transporter, is highly expressed in oxidative fibres (type I fibres) consistent with the role of SLC16A1 in mediating lactate uptake for oxidative metabolism [85]. Its deregulation may be responsible for some of the metabolic impairment in LGMDR1 patients. No previous relation between *Slc16a1* and the Wnt pathway was reported so far, but its downregulation in mouse muscle and in cartilage as well as its upregulation in LGMDR1 patients, where FRZB is overexpressed, suggests a direct interaction in its regulation.

One of the most striking findings was the upregulation of the *Capn3* gene in *Frzb*^−/−^ mice *Soleus* and its upregulation after *FRZB* silencing in human myotubes since no genetic regulatory mechanism of *Capn3* expression has been described so far. *FRZB* upregulation in CAPN3 deficient LGMDR1 patients was already described [10], but the reciprocal regulation has not been reported. However, the increase in *Frzb* expression has been discarded as a beneficial compensatory mechanism since silencing of the gene increased several proteins that were upregulated in LGMDR1 patients [16]. These findings could be interesting not only for LGMDR1 patients, but for dysferlinopathy and titinopathy patients in whom a secondary reduction of *CANP3* has been described [86, 87].

## Conclusion and limitations

In summary, the result presented here confirm a role for *Frzb* in the regulation of *Rora*, *Slc16a1*, *Tfrc*, and *Capn3* genes, which is of interest in understanding molecular changes observed in LGMDR1 patients. However, the studies and results failed to demonstrate a clinical correlate or clinically meaning effect that can be applied to our understanding of LGMDR1. Our specific in vivo and ex vivo analyses may not have captured all differences between the loss of function and wildtype mice, in particular due to the limitations in experimental design in particular when selecting the age of the mice used in the experiments. Even if not directly clinically revelevant for patients with LGMDR1, the involvement of FRZB in myogenesis was confirmed, since this gene regulates MyoD gene expression and the *Frzb*^*−/−*^ mice show reduced muscle strength and gait abnormalities. However, lack of Frzb did not alter skeletal muscle regeneration capacity and neither induced modifications after exercise, with the caveat that our observations can be dependent on the age of the mice studied. Our data thus uncover new roles for FRZB in muscle and support a specific role for FRZB in patients with LGMDR1.

## Methods

### Animals

*Frzb* knockout (*Frzb*^−/−^) mice were previously generated [22]. C57BL/6 mice were at least in the 19th -20th generation of backcrossing. Wild-type C57Bl/6 mice (WT) were purchased from Janvier (Le Genest St Isle, France). Mice were housed in groups of 4–5 mice in Static micro-insulator cage with Macrolon filter with bedding material, under conventional laboratory conditions (14 h light – 10 h dark; 23+/− 2 °C), with standard mouse chow food (Sniff, Soest, Germany) and water provided ad libitum. All studies were performed with the approval from the Ethics Committee for Animal Research (P034/2016; KU Leuven, Belgium) in accordance with relevant guidelines and regulations. Several groups of mice were used for different experiments, their specific characteristics are available in Table 1.

**Table 1:** Animal experiments: overview, set-up and analysis details

Experiment ID	Experiment details
1. General and weight analysis	* 5 to 8-week-old male and female C57Bl/6 J and *Frzb*^−/−^ mice
	* Primary outcome: 5-week-old mice body weight, Fig. 1a
	* Total sample size: *n* = 29; WT C57Bl/6 J: *n* = 15 and *Frzb*^−/−^: *n* = 14
	* Secondary outcome: 8-week-old mice body weight, Fig. 1b
	* Total sample size: *n* = 31; WT C57Bl/6 J: *n* = 20 and *Frzb*^−/−^: *n* = 11
2. Muscle analysis	* 5 to 6-week-old male and female C57Bl/6 J and *Frzb*^−/−^ mice
	* Primary outcome: Mice muscles’ weight, Fig. 1c
	* Total sample size: *n* = 30; WT C57Bl/6 J: *n* = 16 and *Frzb*^−/−^: n = 14
	* Secondary outcome: Myosin heavy chain composition, Fig. 1d
	* Total sample size: WT C57Bl/6 J: n = 3–2 and *Frzb*^−/−^: n = 3–4
	* Secondary outcome: Hanging time, Fig. 1e
	* Total sample size: n = 16; WT C57Bl/6 J: *n* = 7 and *Frzb*^−/−^: *n* = 9
3. Catwalk analysis	* 8-week-old male and female C57Bl/6 J and *Frzb*^−/−^ mice
	* Total sample size (8-week-old): *n* = 36; WT: n = 20, *Frzb*^−/−^: n = 16
	* Primary outcome: Stand, Fig. 2a
	* Secondary outcome: Swing phase, Fig. 2b
	* Secondary outcome: Step cycle, Fig. 2c
	* Secondary outcome: Stride length, Fig. 2d
4. Chronic exercise protocol	* 5-week-old male and female C57Bl/6 J and *Frzb*^−/−^ mice
	* Total sample size: n = 16; WT: n = 9, *Frzb*^−/−^: n = 7
	* Primary outcome: Mice body weight, Fig. 3a
	* Secondary outcome: *Soleus* CSA and histology, Fig. 3b-c
	* Secondary outcome: *Tibialis anterior* fibre type composition, Fig. 3d
5. Cardiotoxin injection	* 10-week-old male and female C57Bl/6 J and *Frzb*^−/−^ mice
	* Total sample size: *n* = 39; WT: *n* = 19, *Frzb*^−/−^: n = 20
	* Primary outcome: Hematoxylin and eosin stained *Tibialis anterior*, Fig. 4a
	* Secondary outcome: Hematoxylin and eosin stained *Soleus*, Fig. 4b
6. Satellite cell isolation	* 4-week-old male and female C57Bl/6 J and *Frzb*^−/−^ mice
	* Total sample size: n = 14; WT: n = 7, *Frzb*^−/−^: n = 7
	* Primary outcome: Satellite cells and myotubes immunofluorescence analysis, Fig. 5

### Human samples

All participants gave informed consent, using forms approved by the Ethics Committee on the Use of Human Subjects in Research at Donostia University Hospital (ASP-FRZ-2017-01) and all the experiments were performed in accordance with relevant guidelines and regulations. Muscle biopsy specimens were obtained from adult patients with LGMDR1 (genetically confirmed) and healthy donors (Supplementary Table S1). Primary human skeletal muscle cell culture and *FRZB* gene silencing experiments were performed in healthy and LGMDR1 patients’ myotubes as previously described [16].

### Muscle strength and endurance analysis

The four limb hanging test was used to monitor muscle strength and endurance (Treat-NMD Neuromuscular Network (SOP (ID) Number DMD_M.2.1.005)) [88]. Five to six-week-old mice were placed once on a crosslinked wire grid. The grid was inverted and the ‘time to fall’ was monitored.

### Mouse gait analysis

The CatWalk™ XT system (Noldus, CatWalk 7.1, The Netherlands) was used to assess gait and locomotion [24]. Mice were placed on the runway for three consecutive runs. Runs were analysed separately and an average of these three runs was used as an individual value. The following parameters were evaluated: *stand* (paw contact time with the glass plate during the step cycle in seconds), *swing phase* (paw time in the air during the step cycle in seconds), *step cycle* (the sum of stand and swing time in seconds) and *stride length* (distance covered by a paw in mm).

### Treadmill exercise

Six-week-old mice were subjected to a 5-week chronic exercise protocol on a four-lane modular treadmill (Columbus Instruments). The exercised group ran 30 min on a horizontal treadmill at 12 m/min twice a week for 5 weeks [89, 90] after a warm-up exercise consisting in 2 min at 4.2 m/min followed by 8 min at 7.8 m/min. Four WT and 2 *Frzb*^−/−^ mice were included in a non-training control group. All mice weights were monitored every training day and muscles were dissected.

### Cardiotoxin injection

Ten-week-old mice were anesthetized by intraperitoneal injection and 3 μl of 50 μM cardiotoxin (CTX; Latoxan, Portes-lès-Valence, France) was injected into the *Tibialis anterior* and 3 μl of 16.7 μM of cardiotoxin into the *Soleus* [91]. *Tibialis anterior* and *Soleus* muscles were dissected at three days, one, two and four weeks after cardiotoxin injection.

### Histology and immunofluorescence

OCT compound (Tissue-Tek) immersed *Tibialis anterior* and *Soleus* were directly frozen into cold 2-methylbutane (Thermo Fisher Scientific). Frozen muscles were sectioned and stained with hematoxylin and eosin. For immunohistochemistry muscle cryosections were fixed (4% paraformaldehyde (PFA) (Electron microscopy sciences; Hatfield, PA, USA)) followed by permeation (0.3% Triton-X (Sigma-Aldrich; San Luis, MO, USA) in PBS) and blocked (PBS containing 3% bovine serum albumin (BSA) (Biowest; Nuaillé, France) solution). For immunostaining, muscles were incubated at 4 °C overnight with the primary antibodies against alkaline phosphatase, ALP (R&D Systems, AF2910; 10 μg/ml), platelet derived growth factor receptor beta, PDGFRβ (CST, #3169; 1:50), neural/glial antigen 2, NG2 (Millipore, AB5320; 1:500) or alpha smooth muscle actin, α SMA (Abcam, ab5694; 1:500) in a PBS containing 3% BSA solution. Isolated cells were fixed (4% PFA in PBS for 10 min), permeabilized (0.2% Triton-X100 in 1% BSA) and blocked (donkey serum 1:10 in PBS; VWR international, Radnor, PA, USA). Primary antibodies were incubated incubated overnight at 4 °C against monoclonal mouse anti-Ki67 (BD Bioscience, San Jose, CA, USA, 556003; 1:300), polyclonal rabbit anti-MyoD (Santa Cruz biotechnology, SC-760; 1:50), mouse anti-MyHC (Developmental Studies Hybridoma Bank -DSHB- Iowa City, IA, USA, 1:20), mouse anti-myogenin (DSHB; 1:10) and polyclonal rabbit anti-sarcomeric α-actinin (Abcam, ab72592; 1:500) in a PBS containing 0.1% Triton-X100 and 0.5% BSA solution. Secondary antibodies were incubated for 1 h at room temperature; goat anti-rabbit conjugated to Alexa-Fluor 555 (A-21428), and 488 (A-11034), donkey anti-mouse conjugated to Alexa-Fluor 594 (A-21203) and donkey anti-goat conjugated to Alexa-Fluor 488 (A-11055, Thermo Fisher Scientific; 1:500). Nuclei were visualized with 10 μg/ml containing Hoechst (Sigma-Aldrich) solution. Fluor Save reagent (Millipore) was used as mounting medium. Muscle structure was analysed using a Nikon 80i microscope and the NIS-Element software. For satellite cell immunofluorescence analysis, the percentage of positive nuclei was counted in randomly selected 6 fields of view. Fusion index was calculated in myotubes as the percentage of nuclei inside myotubes, being MyHC or sarcomeric α-actinin, myotubes markers. Between 5 and 6 field of view were counted for each sample. For fibre type classification SDH enzymatic activity was used [92]. The fibres were assigned to four different groups depending on the intensity (in pixels) value obtained with the ImageJ program (strong< 100, medium-stron 100–150, medium-weak 150–200 and weakly coloured > 200 pixels). All fibres of one section of *Tibialis anterior* per mouse in to the chronic exercise protocol experiment were measured.

### Muscle fibres cross-sectional area

ImageJ software was used to measure fibres cross-sectional area (CSA). One hundred fibres from 4 different fields of view were measured.

### Mouse primary cells

#### Satellite cells

*M*urine satellite cells from 4-week-old mice were isolated from *Gastrocnemius*, *Tibialis anterior*, *Quadriceps* and *Biceps* as previously described [93]. Cells were plated in triplicate and at confluency, the differentiation was induced by switching medium to DMEM high glucose supplemented with 2% horse serum (Gibco-Thermo Fisher Scientific) and 1 mM (100 mg/ml) sodium pyruvate, 100 U/ml penicillin and streptomycin, 2 mM L-glutamine. Cells were incubated at 37 °C, 5% CO_2_, 5% O_2_.

#### Mesoangioblasts

Mesoangioblasts (MABs) were isolated from *Gastrocnemius*, *Tibialis anterior*, *Quadriceps* and *Biceps* explant culture, by Fluorescence Activated Cell Sorter (FACS; BD FACS ARIA III) for alkaline phosphatase (ALP) + cells (R&D Systems-Biotechne, Minneapolis, MN, USA) from 5 week-old mice as previously described [94]. Flow cytometry analysis of the ALP+ fraction was carried out in 3 WT and 7 *Frzb*^−/−^ samples. Protein tyrosine phosphatase receptor type C (CD45), platelet and endothelial cell adhesion molecule 1 (CD31) and platelet derived growth factor receptor alpha (PDGFRα or CD140α; Thermo Fisher Scientific) cell surface proteins presence were analysed by flow cytometry (FACs; BD Canto AIG) and analysed by BD FACSDiva software.

### RNA extraction

Muscle samples were homogenised in a Tissue Lyser mixer-mild disruptor (QIAgen, Hilden, Germay) in Trizol (QIAzol® lysis reagent, QIAgen). Total and small RNAs were purified using miRNeasy mini kit (QIAgen) following the manufacturer’s instructions.

### Gene expression analysis

RNA was reverse-transcribed to cDNA using High Capacity cDNA Reverse Transcription Kit (Applied Biosystems; Foster City, CA, USA) according to the manufacturer’s instructions. For gene expression analyis Taqman single assays (Thermo Fisher Scientific; Walthman, MA, USA) and custom-designed SYBR green panels (Bio-Rad, Hercules, CA, USA) were used. As TaqMan probes the following genes were studied; myogenic or skeletal muscle specific markers, *Pax7* (Mm01354484_m1), *Myh3* (Mm01332463_m1), *Myod* (Mm00440387_m1) and *Myog* (Mm00446194_m1). *Fbx32* (Mm00499523_m1) and *Murf1* (Mm01185221_m1) for muscular atrophy. *Adipoq* (Mm00456425_m1) and *Pparg* (Mm01184322_m1) for adipose tissue infiltration measurement. *Capn3* (Mm00482985_m1), *Ky* (Mm01224823_m1) and *Park2* (Mm00450186_m1) were selected from a list of deregulated genes in *Capn3* knockout (C3KO) mice [25]. *Gapdh* (Mm99999915_g1) and *Tpb* (Mm00446973_m1) were used as housekeeping. The entire list of the custom-designed SYBR green panels can be found as Supplementary Table S2 online. a) muscle specific genes, b) deregulated genes in LGMDR1 patients’ muscles [10, 95], c) deregulated genes in *Frzb*^−/−^ mice articular cartilage and LGMDR1 patients’ muscles [23, 26] d) genes coding for proteins participating in Wnt signalling pathway. *Gapdh* and *Tpb* were used as housekeeping. In human origin myotubes *FRZB* (Hs00173503_m1), *MYOD* (Hs00159598), *MYOG* (Hs01072232) and *CAPN3* (Hs00181057_m1) genes were analysed. *GAPDH* (Hs99999905_m1) was used as housekeeping. For RT-QPCR analysis the CFX384 Touch PCR System (Bio-Rad) and CFX Manager Software was used. Relative gene expression levels between WT mice and *Frzb*^−/−^ mice muscles were calculated using the 2^-ΔΔCT^ method.

### Statistical analysis

Data are presented as mean and SEM, or as individual data points. Statistical analyses were performed where appropriate with GraphPad Prism software (version 8) or R Studio (Version 1.0.15) for analyses with multiple within-subject variables, using the aov_car function from the afex package. Data distribution was evaluated based on parameter characteristics, QQ plots and graphs of the residuals. *T*-tests or ANOVA-tests were applied. Data are reported with estimates of differences of means between groups (95% confidence intervals). Datasets with within-subject variables (repeated measurements) were analysed with 2-way ANOVA or a general linear model (GLM) in case of missing data. When different groups were compared by ANOVA or GLM tests, in some set-ups pair-wise *t*-tests were subsequently performed applying a Bonferroni correction for multiple comparisons to control for Type I errors in rejecting the null hypothesis.

## Supplementary information

**Additional file 1: Table S1.** Human Tissue Samples. **Table S2.** Custom-designed SYBR green panel’s gene selection (Bio-Rad).

## Data Availability

The datasets used and/or analysed during the current study are available from the corresponding author on reasonable request. All data generated or analysed during this study are included in this published article (and its supplementary information files).

## Abbreviations

Adipoq: Adipose most abundant gene transcript 1 protein
ALP: alkaline phosphatase
BSA: Bovine serum albumin
C3KO: Capn3^−/−^ mouse
CAPN3: Calpain 3
CSA: cross-sectional area
CTX: Cardiotoxin
DMD: Duchenne Muscular Dystrophy
Fasn: Fatty acid synthase
Fbx32: F-Box protein 32
FP: Front paw
FRZB: Frizzled Related Protein
FSH: Facioscapulohumeral
HP: Hind paw
Ky: Kyphoscoliosis peptidase
LGMD2A: Limb girdle muscular dystrophy type 2A, calpainopathy
LGMD2B: Limb girdle muscular dystrophy type 2B, dysferlinopathy
LGMD2I: Limb girdle muscular dystrophy type 2I
LGMD2L or LGMDR12: Limb girdle muscular dystrophy type 2 L, anoctaminopathy
LGMDR1: Limb girdle muscular dystrophy R1 calpain 3-related
MABs: Mesoangioblasts
MuRF1: Muscle-specific RING finger protein 1
MyHC: Myosin heavy chain
Myod: Myogenic Differentiation 1
Myog: Myogenin
NG2: Neural/glial antigen 2
Park2: Parkin
RBR E3: Ubiquitin protein ligase
Pax7: Paired box 7
PDGFRβ: Platelet derived growth factor receptor beta
PFA: Paraformaldehyde
Pparg: Peroxisome proliferator activated receptor gamma
Rora: RAR Related Orphan Receptor A
Slc16a1: Solute Carrier Family 16 Member 1
Tfrc: Transferrin Receptor
WT: Wild type
α SMA: Alpha smooth muscle actin
