# Supplementary material for: Frizzled related protein deficiency impairs muscle strength, gait and calpain 3 levels
Source: Orphanet J Rare Dis. 2020 May 24;15:119. doi: 10.1186/s13023-020-01372-1 (PMC7245871; doi:10.1186/s13023-020-01372-1)
Supplement: Supplementary file 1 — Additional file 1: Table S1. Human Tissue Samples. Table S2. Custom-designed SYBR green panel’s gene selection (Bio-Rad). [file 13023_2020_1372_MOESM1_ESM.pdf]

Supplementary Table S1. Human Tissue Samples.

Group	Age	Gender	Tissue Origin	Ambulation status	*CAPN3* mutations	
					Mutation 1	Mutation 2
LGMDR1	19	Male	*Biceps*	Ambulant	p.(His690ArgfsX9)	p.(His690ArgfsX9)
LGMDR1	28	Male	*Deltoid*	Ambulant	p.(Lys254Glu)	p.(Pro637HisfsX25)
LGMDR1	29	Male	*Deltoid*	Wheelchair bound	p.(Lys254del)	p.(X822Leuext62X)
Healthy	23	Male	*Biceps*			
Healthy	14	Male	*Quadriceps*			
Healthy	36	Female	*Biceps*			

Supplementary Table S2. Custom-designed SYBR green panel's gene selection (Bio-Rad).

	Gene name	Gene symbol	Amplicon context sequence	Amplicon lenght (bp)
	Muscle specific genes			
1	Myosin, heavy polypeptide 1, skeletal muscle, adult	*Myh1*	CAAGTGAGTGAGCTGAAGACCAAGGAGGAGGAACAGCAGCGGCTGATCAATGAGCTGACTGCGCAGAGGGGGCGTCTGCAGACGGAGTCAGGTGAATACTCACGCCAGCTAGA	113
2	Myosin, heavy polypeptide 2, skeletal muscle, adult	*Myh2*	AACACGAGAGACGAGTGAAGGAGCTTACTTACCAGACAGAAGAAGACCGAAAAAATATTCTCAGGCTTCAGGATTTGGTGGATAAACTCCAGGCAAAAGTGAAATCTTACAAGAGACAAGCTGAGGAGGCTGAGGAACAATCCAACACAAATCTATCCAAGTTCCG	166
3	Myosin, heavy polypeptide 4, skeletal muscle	*Myh4*	CAGAAATCCGGGTTGAAGACTCTGGCTTTCCTATTTTCTGGGGGACAAGCTGCGGAAGCAGAGGGCGGCGGTGGAAAGAAAGGTGGCAAGAAGAAGGGTTCTTCTTTCCAGACCGTGTCAGCTCTCTTCAGGGAGAATTTAAATAAGCTGATGACCAACTTGAAGAGCACCCACCCCCACTTTGTCAGATGCCTCATTCC	200
4	Paired box gene 7	*Pax7*	ACTCGGGTTGCTAAGGATGCTCATGACCTGAGGAGACAGGCCATTGCTGACAGGGTTCAT	60
	Derregulated genes in LGMDR1 patients' muscles			
5	Calpain 3	*Capn3*	ATTCATCCTCCGAGTCTTCTCCGAAAAGAGGAATCTCTCTGAGGAAGCTGAAAATACAATCTCTGTGGATCGG	73
6	Calpain 6	*Capn6*	GTCCTTCTGTTGCAGTGACATGATGACTTTATGGCCATCCTCGGGCACAGTGAAAATGTACTGAGGATTCTGCAAGAAGGTATCACGGTTGTTATAGCAACCTCCTGATCGGTTCAT	117
7	Collagen, type I, alpha 1	*Col1a1*	GCATGGCCAAGAAGACATCCCTGAAGTCAGCTGCATACACAATGGCCTAAGGGTCCCCAATGGTGAGACGTGGAAACCCGAGGTATGCTTGATCTGTATCTGCC	104
8	Docking protein 5	*Dok5*	AACTTTCACTCCCTCAGTCTCTGTCCTGACCCTCCTCCCCACATCCCTCCTCGAAGCCACTCAGGGTGCCGAGCGCACGCTGGGGGCAGCAGGGTCACTGTCTGTCTGGGATGGCTTCCAATTTTAATGACATAGTGAAGCAGGGGTACGTGAGGATCCGGAGCAGACGCCTAGGGATTTATCAACGATGCTGGTTAGTG	200
9	Four and a half LIM domains 1	*Fhl1*	GTGCTTTGACAAGTTCTGCGCCAACACCTGCGTGGACTGCCGCAAGCCCATAAGCGCTGATGCCAAGGAGGTGCATTATAAGAATCGCTACTGGCACGACAACTGCTTCCGCTGTGCCAAGTGCCTTCACCCCTTGGCCAGTGAGACCTTTGTGTCCAAGGATGGCAAGATCCTGTGCAACAAGTG	186
10	Integrin beta 1 binding protein 2	*Itgb1bp2*	AAGTTCACTTCAGGAGCAAAAACCTCTAAATACAATTCCAAAGTCAGCAGAGACCTTGTTCCGAGAAAGGCCTAAGTCTGAGATGCCTCCCAAACTGCTACCACTTCTT	109
11	Myosin, light polypeptide 6B	*Myl6b*	TCTACACGTCGGGACTTCAGCTCCTCGTTTTTGGGGTTCCCCAGGACCTTGAGCACCTCGGCGTTGGTAGGGTTCTGGCCCAGGGCCCTCATCAGGTCCCCACACTGGCTGTACAGGATCTTGCCATCACCTACTCGGTCAAAC	144
12	Myomesin family, member 3	*Myom3*	GGTGTCTGAATGTGCATAGAGACTGCCTGACCCTGACCTGGGTCCCGCCCAGCGACACCCGGGGCAGCACCATCACTGGCTATTCCATTGAGATGTGCCAGGGTGATTCGGAGGAGTGGA	120
13	Myotilin	*Myot*	CTCAGTTCTATATTACCGTCTCAACCCGATTACTGTAACAGTAAAATCCCATCCACTGTGGACTCCAACTATCAACAATCCTCAGTTAACCAACCTG	97
14	Very low density lipoprotein receptor	*Vldlr*	GCTTCTGTAGGACACACATACCCAGCAATATCAGTTGTAAGCACAGATGATGATCTGGCTTGAGTTCTGAAC	72
	Derregulated genes in Frzb$^{-/-}$ mice articular cartilage and in LGMDR1 patients' muscles			

16	Asporin	*Aspn*	GTTGTTCACTGCTCTGATCTAGGTCTGACATCGGTTCCAAACAACATTCCATTTGA TACTCGAATGGTTGACCTTC	76
17	Collagen, type III, alpha 1	*Col3a1*	GTGGAACCTGGTTTCTTCTCACCCTTCTTCATCCCACTCTTATTTGGCACAGCAGT CCAACGTAGATGAATTGGGATGCAGCCACCTTGGTCAGTCCTATGAGTCTAGAG ATGTCTGGAAGCCAGAACCATGTCAAATATGTGTCTGTGACTCAGGA	158
18	Collagen, type V, alpha 1	*Col5a1*	GTTGCCTACCGAGTCTCTAAAGATGCACAGCTCAGCATGCCCACCAAGCAGCTGT ACCCTGAGTCTGGTTTTCCCGAGGACTTCTCCATCCTGACAAC	98
19	Collagen, type XV, alpha 1	*Col15a1*	GTCCCTCTGGAAATGATGAAGGGGAGAAAGGGTGAACCTGGAATCCATGGTGC ACCGGGACCCATGGGACCCAAAGGACCACCAGGACACAAAGGAGAGTTTGGC	105
20	Collagen triple helix repeat containing 1	*Cthrc1*	CCATCGAAGCCATCATCTATCTGGACCAAGGAAGCCCTGAGTTAAATTCAACTAT TAATATTCATCGTACTTCCTCTGTGGAAGGACTCTGTGAAGGGATTGGTGCTGGA TTGGTAG	117
21	Catenin (cadherin associated protein), beta 1	*Ctnnb1*	ACAGCTCCCCTGACAGAGTTACTCCACTCCAGGAATGAAGGCGTGGCAACATAC GCAGCTGCTGTCCTATTCCGAATGTCTGAGGACAAGCCACAGGATTACAAGAAG CGGCTTTCAGTCGAGCTGACCAGTTCCCTCTTCAGGACAGAGCCAATGGCTTGG AATGAGACTGCAGATCTTGGACTGGACATTGGTGCCCAGGGAGAAGCC	180
22	E2F transcription factor 8	*E2f8*	CAATGTCATACAGCCTCCTAATTTTTGTTTTATACTTGCTTTTATCCAGATCTTCCA CGTGGTCTTCCCCAATTAAAATCTTGGCAGCAATTTCCAGGCTCACTATCTGAGG CGTCGAC	119
23	Fatty acid synthase	*Fasn*	AGACGCCAGTGTTCGTTCCTCGGAGTGAGGCTGGGTTGATACCTCCATCCACAAT TGCTTCATAGCTGACTTCCAACAG	79
24	Fibronectin 1	*Fn1*	CAGAGTCGCACTGGTAGAAGTTCCAGGAACTTGGAACTGTAAGGGCTCTTCGTC GGTGCCAACTGGTTGGCATGAAATGATGTACTCAGAACTCTCCTGGAACG	104
25	Frizzled-related protein	*Frzb*	GCGGTCACATCATGACATTTCATCTTTACCTCTTTAACTTTAGCCCGGATGACATA GTTGTAATTGTTCCGGAAATAGGTCTTCTGTGTAGCTCTGACA	99
26	Insulin-like growth factor 1	*Igf1*	GGTGGATGCTCTTCAGTTCGTGTGTGGACCGAGGGGCTTTTACTTCAACAAGCCC ACAGGCTATGGCTCCAGCATTCGGAGGGCACCTCAGACAGGCATTGTGGATGAG TGTTGCTT	117
27	Mesoderm specific transcript	*Mest*	CTCTGCACTCATGGAAGACTTCTGGCAAGTTTTTCACCTACAAAGGCCTACGCAT CTTCTACCAAGTTAGATTTTGGTGTCGTCAAGGCTGCCTTGCATGCAGTTTGCCTC ATTTCTCATTTTCCTCCTAGATTCTGTCGG	141
28	Myelocytomatosis oncogene	*Myc*	CTAGTGCTGCATGAGGAGACACCGCCCACCACCAGCAGCGACTCTGAAGAAGAG CAAGAAGATGAGGAAGAAATTGATGTGGTGTCTGT	89
29	RAR-related orphan receptor alpha	*Rora*	TACAGAAGAACCACCGAGAAGATGGAATTCTAACCAAGCTAATATGCAAGGTGT CTACGTTAAGAGC	67
30	Sema domain, immunoglobulin domain (Ig), short basic domain, secreted, (semaphorin) 3C	*Sema3c*	CTCCACAGGCATCTATCAAGTGGTTGCTGCAGAAAGACAAAGACAGGAGGAAG GAGGTTAAACTGAACGAGCGCATTATAGCTACTTCCCAAGGACTACTGATTCGCT CTGTTCAAGA	118
31	Solute carrier family 16 (monocarboxylic acid transporters), member 1	*Slc16a1*	CTGTAACACAGTACAGGAACTTTACTTGTGCATTGGTGTTATTGGAGGTCTTGGG CTTGCTTTCAACTTGAACCCAGCTCTGACTATGATTGGCAAGTAT	100
32	Sorbin and SH3 domain containing 1	*Sorbs1*	AATGTCTTGGTGACTCTGAATCTTTAGTGGAAGCTGAGCTGGAAGGTCGCTTGA GCCCACTGAGGCCCTGAAGAGGGATGTCGCCACCTTCCAAGACACTCTTATAGA TCTGCCTCT	117
33	Transferrin receptor	*Tfrc*	AGCCAGATCAGCATTCTCTAACTTGTTTGGTGGGGAACCATTGTCATACACCCGG TTTAGCCTTGCTCGGCAAGTAGATGGAGATAACAGTCATGTGGAGATGA	104
	Genes coding for proteins participating in Wnt signalling pathway			
34	Low density lipoprotein receptor-related protein 5	*Lrp5*	TAGTCACTGTCACACACATCTGTGCTGCACGGTGTTGTTGGGGGTGCCATACCTC GAATGACGTAGGGCCTGTATGGTCTAGCGGTGG	88
35	Low density lipoprotein receptor-related protein 6	*Lrp6*	TAGGAGCATAGTCACTGTCACAGACATCAGTGCTGCAGGGTGTGGTGGGCGGT GCAAAGTGCCGGTAGCTGTACGGCCTATAGCTGTAGGACCTATGTGTGGAAGGA CTGTTG	113
36	Wingless-related MMTV integration site 8A	*Wnt8a*	CGAAGAGTGTAAGTTCCAGTTTGCCTGGGAACGGTGGAATTGTCCTGAGCATGC TTTTCAGTTTTCAACCCACAACAGGCTGCGAGCTGCCACGAGAGAGACATCCTTC ATTCATGCC	118
37	Wingless related MMTV integration site 8b	*Wnt8b*	GTGCGTTCTTCTAGTCACTTGTGTCCTTCACCGCAGCCACGCCTGGTCAGTGAAC AATTTTCTGATGACCGGTCCAA	77
	Housekeeping genes			
38	Glyceraldehyde-3-phosphate dehydrogenase	*Gapdh*	AACCTGGTCCTCAGTGTAGCCCAAGATGCCCTTCAGTGGGCCCTCAGATGCCTGC TTCACCACCTTCTTGATGTC	75
39	TATA box binding protein	*Tbp*	GGAGAATCATGGACCAGAACAACAGCCTTCCACCTTATGCTCAGGGCTTGGCCT CCCCACAGGGCGCCATGACTCCTGGAATTCCCATCTTTAGTCCAATGA	102
